# Editorial: Health and corporate/urban sustainability

**DOI:** 10.3389/fpubh.2025.1603877

**Published:** 2025-04-14

**Authors:** Jianfu Shen, Xunpeng Shi, Eddie C. M. Hui

**Affiliations:** ^1^Hong Kong Polytechnic University, Kowloon, Hong Kong SAR, China; ^2^University of Technology Sydney, Sydney, NSW, Australia; ^3^City University of Hong Kong, Kowloon, Hong Kong SAR, China

**Keywords:** health, corporate sustainability, urban sustainability, innovation, policy

The intersection of health, corporate practices, and urban development has emerged as a critical frontier in addressing global sustainability challenges. This Research Topic, Health and Corporate/Urban Sustainability, seeks to bridge these gaps by presenting interdisciplinary insights into how health—both at individual and community levels—shapes sustainable development trajectories. The 12 articles in this Research Topic underscore that prioritizing health is not merely a moral imperative but a strategic lever for fostering economic stability, environmental sustainability, and societal wellbeing. This research can potentially inspire transformative change in our approach to sustainability.

## Health as a catalyst for corporate sustainability and innovation

Corporate sustainability is increasingly intertwined with employee health and safety, as evidenced by several contributions. Nawata reveals that physical and mental health conditions, such as hypertension, stress, and inadequate work-life balance, significantly correlate with long-term absenteeism, imposing financial burdens on firms. These findings reinforce the need for workplace interventions targeting preventive healthcare and mental wellbeing to enhance productivity. On the other hand, Wang and Yuan demonstrate how digital platforms amplify corporate social responsibility (CSR) by facilitating environmental innovation. Their study highlights a 55.31% improvement in policy enforcement when firms adopt digital tools, underscoring technology's role in aligning CSR with public health outcomes.

The nexus between policy and corporate innovation in biopharmaceutical industry is further explored by Xia and Jia, who identify industry-university-research (IUR) collaborations as a driver of biopharmaceutical innovation. Their nuanced analysis cautions that government subsidies, while boosting R&D inputs, may inadvertently stifle innovation quality—a paradox demanding policy recalibration. Meanwhile, Wu et al. illustrate how economic policy uncertainty spurs Chinese pharmaceutical firms' innovation, particularly through financialization and executive incentives. These studies advocate for adaptive governance frameworks that balance risk, innovation, and corporate stakeholder welfare.

## Urban health: resilience, equity, and recovery

Urban sustainability hinges on equitable health systems and resilient infrastructure. Zhang and Deng emphasize this by linking internal migrants' health to economic resilience in the Yangtze River Delta, China. Their analysis reveals that migrants' health boosts labor participation, enhancing cities' capacity to withstand crises—a relationship moderated by access to public health services. This aligns with Makoni et al.'s advocacy for community-led monitoring, whereby Zimbabwe leverages local engagement to reduce HIV's stigmas and improve service delivery. Such grassroots approaches exemplify how decentralizing health governance can strengthen urban resilience.

The COVID-19 pandemic laid bare systemic vulnerabilities in the healthcare system, prompting Zhang et al. to propose a loosely coupled process management framework for epidemic prevention. By integrating digital systems with adaptable workflows, their model mitigates healthcare strain—a lesson critical for future pandemic preparedness. Further, Linghu et al. demonstrate that information and communication technology (ICT) development in China enhances carbon emission efficiency by 0.11% per 10% increase in internet penetration, yielding public health co-benefits through cleaner energy and innovation. These articles collectively argue for technology-driven, inclusive urban planning to harmonize environmental and health goals, empowering our readers with the knowledge and tools they need.

## Policy, innovation, and regional pathways

Regional case studies offer valuable lessons for global sustainability. Lin et al. uncover that income inequality in rural China exacerbates health disparities, mitigated by education and health awareness. Their work underscores the need for targeted policies to address socio-economic determinants of health. Similarly, Meng et al. analyse Guangxi's post-pandemic tourism-urbanization-environment nexus, advocating for digital transformation and green practices to revive tourism sector while safeguarding environmental health.

At the macro-economic level, Fan et al. resolve longstanding debates through a meta-analysis of 479 studies, confirming a strong positive correlation (*r* = 0.429) between health insurance and economic performance. Their findings reveal that public insurance systems in developed nations yield greater economic returns, urging policymakers to tailor healthcare financing to local contexts. Complementing this, Li and Ma attribute 3.9% growth in comprehensive total factor productivity to digital economy advancements in China, driven by technological innovation and education—a blueprint for emerging economies.

## Toward integrated sustainability

This Research Topic has advocated for a paradigm shift: viewing health not as a siloed sector but as a linchpin of holistic sustainability. Corporate strategies must integrate employees' wellbeing with environmental and governance goals, while urban policies should prioritize equitable health access to bolster economic resilience. Technological innovation—from digital platforms to green ICT—emerges as a cross-cutting enabler, yet its success hinges on contextual adaptation and inclusive designs.

As nations navigate post-pandemic recovery and climate challenges, this Research Topic emphasizes the urgency of embedding health into the sustainability framework. Policymakers, corporate leaders, and urban planners are urged to adopt synergistic approaches that recognize health as both a driver and outcome of sustainable development. The practical implications of this research can empower these stakeholders to make informed decisions that will shape the future of our world.

This editorial synthesizes contributions to the Research Topic “*Health and Corporate/Urban Sustainability*.” We extend gratitude to all authors and reviewers for advancing this critical dialogue. Their collaborative efforts have created a community of scholars and practitioners dedicated to advancing health and sustainability.

